# Harnessing the microbiome to control plant parasitic weeds

**DOI:** 10.1016/j.mib.2019.09.006

**Published:** 2019-06

**Authors:** Raul Masteling, Lorenzo Lombard, Wietse de Boer, Jos M Raaijmakers, Francisco Dini-Andreote

**Affiliations:** 1 Department of Microbial Ecology, Netherlands Institute of Ecology (NIOO-KNAW), Wageningen, The Netherlands; 2 Institute of Biology, Leiden University, Leiden, The Netherlands; 3 Westerdijk Fungal Biodiversity Institute, Utrecht, The Netherlands; 4 Chair Group Soil Biology, Wageningen University and Research (WUR), Wageningen, The Netherlands; 5 Department of Plant Science, The Pennsylvania State University, University Park, PA, USA; 6 Huck Institutes of the Life Sciences, The Pennsylvania State University, University Park, PA, USA

## Abstract

Microbiomes can significantly expand the genomic potential of plants, contributing to nutrient acquisition, plant growth promotion and tolerance to (a)biotic stresses. Among biotic stressors, root parasitic weeds (RPWs), mainly of the genera *Orobanche*, *Phelipanche* and *Striga*, are major yield-limiting factors of a wide range of staple crops, particularly in developing countries. Here, we provide a conceptual synthesis of putative mechanisms by which soil and plant microbiomes could be harnessed to control RPWs. These mechanisms are partitioned in direct and indirect modes of action and discussed in the context of past and present studies on microbe-mediated suppression of RPWs. Specific emphasis is given to the large but yet unexplored potential of root-associated microorganisms to interfere with the chemical signalling cascade between the host plant and the RPWs. We further provide concepts and ideas for future research directions and prospective designs of novel control strategies.

## Highlights

• Plant microbiomes have an unexplored potential to control root parasitic weeds.
• Understanding the mechanisms by which microbes can control parasitic weeds is largely elusive.
• Members of the root microbiome can interfere with host-parasite chemical communication.
• Direct and indirect modes of action can work synergistically in microbe-mediated weed control.

**Current Opinion in Microbiology** 2019, **49**:26–33

This review comes from a themed issue on **Environmental microbiology**

Edited by **Roeland Berendsen** and **Klaus Schlaeppi**

For a complete overview see the Issue and the Editorial

Available online 23rd October 2019

https://doi.org/10.1016/j.mib.2019.09.006

1369-5274/© 2019 The Authors. Published by Elsevier Ltd. This is an open access article under the CC BY license (http://creativecommons.org/licenses/by/4.0/).

## Introduction

The economically most important root parasitic weeds (RPWs) belong to the family Orobanchaceae, encompassing the genera *Orobanche,Striga* and *Phelipanche*. These RPWs have a hidden but devastating effect on host plants as a large part of its life cycle occurs belowground. Once the parasite emerges aboveground, the adverse impact on crop productivity has already taken place. *Striga* species, also known as witchweeds, are widely distributed in Sub-Saharan Africa, India and Southeast Asia [1], affecting cereal crops such as maize, rice, millets, sorghum and the legume cowpea. *Striga* causes yield losses up to 80%, often resulting in field abandonment by local farmers. For *Striga hermonthica* it has been estimated that 50–300 million hectares of field soils in Africa are currently infested [2]. In addition to *Striga* spp., also the broomrapes *Phelipanche* and *Orobanche* are widely distributed and their hosts are not limited to cereals and legumes, but also comprise Solanaceae(e.g. tomato, tobacco), Asteraceae (e.g. sunflower), and Cucurbitaceae (e.g. watermelon). They substantially affect crop production in Western Africa, the Mediterranean area but also occur in Australia, America and Asia. For *Orobanche crenata,* legume crop losses of up to 100% have been reported in Morocco, Portugal, Spain and Syria [2].

Despite their wide geographic distribution and host range, the RPW’s life cycles and infection strategies have common traits. For obligate RPWs, seed germination relies on host-derived signals released by the roots, in particular the strigolactones. The primary eco-evolutionary role of these multi-functional phytohormones is to initiate, under low nutrient conditions, a symbiotic association with arbuscular mycorrhizal fungi (AMF) [3]. Hence, obligate RPWs hijack these signals for infection, repurposing this ancient beneficial signalling mechanism [4]. The germination signal is perceived by the RPWs via strigolactone receptors [5], but the downstream signalling is not yet fully resolved [6]. Following seed germination, an important second step in root infection by RPWs is haustoria formation. Also here the underlying chemistry has received considerable attention and various haustorium-inducing factors have been identified, including quinones (e.g. 2,6-dimethoxy-1,4-benzoquinone), phenolic compounds (e.g. syringic acid, vanillic acid, vanillin), and anthocyanins (e.g. peonidin, pelargonidin) [7,8]. Other key stages of the life cycle that are promising targets for control include the seed bank in soils and the production of new seeds [9]. Current control strategies include breeding for host resistance, cultural methods such as hand weeding and alternative cropping practices, and chemical control. Each of these strategies is not singularly effective and not always available to smallholder farmers [9]. Hence, a systems approach is needed to provide effective and sustainable control of RWPs.

In this opinion article, we provide a conceptual framework to explore the yet-untapped potential of soil and root-associated microbes to interfere with the chemical signalling cascade and to induce physiological and phenotypic changes in the host plant to suppress RPWs. We discuss direct and indirect modes of action in the ecological context of the tripartite interaction between host, parasite and microbiome. We argue that understanding the intricate eco-evolutionary, chemical and genetic mechanisms operating at the root-soil interface constitutes an essential step towards developing new integrated strategies to mitigate the adverse impacts of RPWs on crop production.

## Microbe-mediated mechanisms of root parasitic weed control

**Figure 1:**
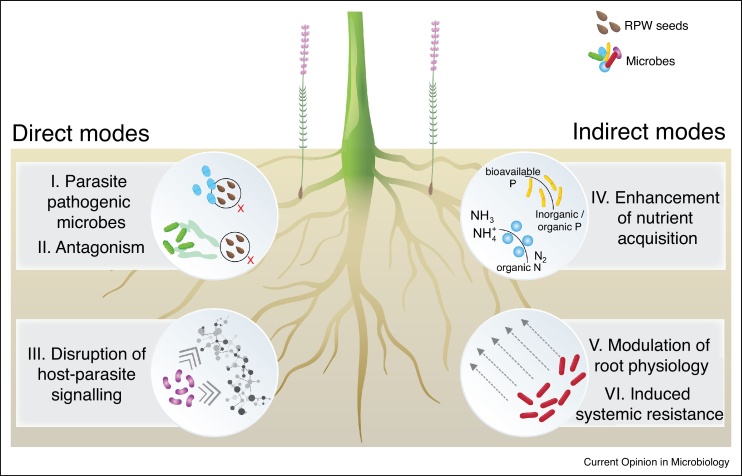
Microbe-mediated mechanisms for root parasitic weed (RPW) control. The conceptual figure depicts examples of direct modes of action that target the RPWs by hindering or disrupting the RPW’s life-cycle. Indirect modes of action comprise those in which microbes affect the soil nutrient pool bioavailable to the plant, affect plant physiology or induce local and systemic resistance against RPW infections.

**Figure 2:**
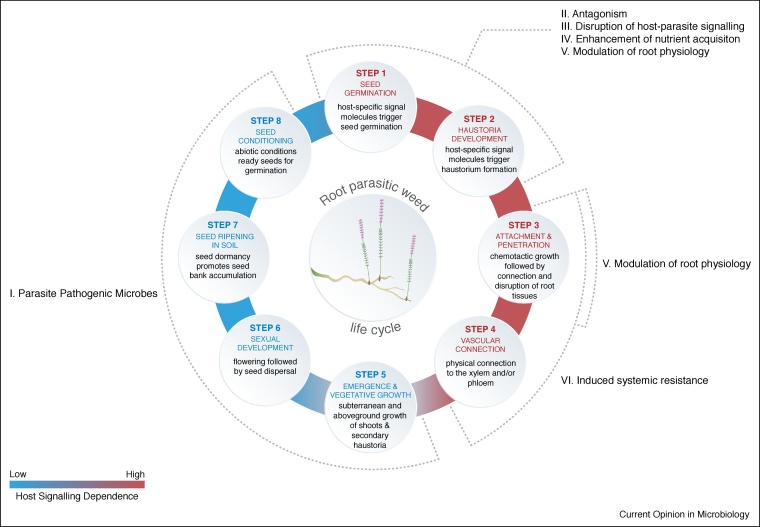
Signalling and life cycle of root parasitic weeds. **(1)** Host plant roots release signalling molecules (i.e. strigolactones) that induce the germination of root parasitic weed (RPW) seeds in the root-soil interface. **(2)** After germination, the parasite forms radicles and haustoria, the formation of which are induced by molecules known as haustorium-inducing factors. **(3)** The haustorium connects to and penetrates host roots reaching the vascular tissues. **(4)** RPWs establish a vascular connection with the xylem and/or xylem and phloem (this is dependent on the photosynthetic capability of the RPW species) in order to syphon water and photosynthates from the host plant. **(5)** Once a functional vascular connection is established, the RPW undergoes vegetative growth, followed by emergence from the soil; in some cases, secondary haustoria are formed allowing for additional connections with the host(s). **(6)** After weeks of vegetative growth, the RPWs flower and set seeds. **(7)** The newly formed RPW seeds are deposited in the soil, where they can remain dormant (i.e. RPW seed bank), **(8)** Before being able to respond to host signals, RPW seeds require a pre-conditioning stage that is provided by specific abiotic soil conditions, that is, moisture and temperature. Note that for facultative RPWs **step 1** is not dependent on host-specific signal molecules, as it is for obligate RPWs, but can be triggered endogenously. The general life cycle of a root parasitic weed (RPW). Schematic presentation of the different steps of an obligate RPW’s life cycle and its dependency on host signals. The warmth of the colours (blue to red) in the outer circle indicates how dependent the RPW is on signalling molecules from the host to serve as cues for its development and to complete its life cycle. Microbe-mediated mechanisms and their most preferred timing to control RPWs are indicated along the dotted line.

Microbes can directly and indirectly interfere in the RPW’s life cycle, either by deterring the parasite or by triggering processes that impair infection of the host roots (Figure 1). Direct modes of action are those in which the microbe or microbiome interact directly with the parasite: these include (*1*) pathogenicity towards the RPW, (*2*) antagonism towards RPWs via secondary metabolites, and (*3*) interference with host-parasite signalling. We refer to indirect modes of action as those in which the microbe or microbiome affect the parasite through interactions with the host and/or local environment. These modes of action include *(4)* enhancement of nutrient acquisition by the host, in particular phosphorous (P) and nitrogen (N), *(5)* modulation of host root physiology, that is, alteration of exudation or root architecture, and *(6)* induced systemic resistance (ISR). Importantly, these different mechanisms are not mutually exclusive and likely work in sequence, simultaneously or even synergistically during the RPW life cycle (Figure 2).

## Direct modes of action

### RPW pathogens

There are two important considerations with respect to the use of pathogens to control RPWs: *(1)* host specificity of the pathogen, and *(2)* stage of the RPW’s life cycle affected by the pathogen. One of the most studied RPW pathogens is the fungus *Fusarium*, with ca. 15 species tested against parasitic weeds from the genera *Orobanche*, *Striga* and *Phelipanche* [10]. Only *Fusarium oxysporum* f. sp. *strigae* was shown to be specific to *S. hermonthica*, with the exception of some solanaceous plants which also can be colonised by this fungus [11]. In a consortium consisting of three strains of *F. oxysporum* f. sp. *strigae* (called Foxy T14), the overproduction of tyrosine, leucine and/or methionine (due to metabolic imbalances and inhibitory feedbacks [12]) was significantly related to reduced emergence of *Striga* and consequently increased yields of maize [13^••^]. Also other fungal species including *Alternaria*, *Aspergillus* and *Verticillium* were reported as pathogens of *Striga* spp., with emphasis on *S. hermonthica,* resulting in a significant reduction of RPW emergence and biomass [10]. For *O. crenata*, the fungus *Ulocladium atrum* was shown to infect vegetative structures, such as shoots and tubercules, thus hindering RPW infection and development [14]. An excellent example of a pathogen acting at early stages of RPW development is the fungus *F. oxysporum* f. sp. *orthoceras,* which colonises seeds of *Orobanche cumana*, and act by dissolving the seed endosperm and metabolizing cytoplasmic compounds [15]. Next to fungi, several bacterial genera such as *Bacillus*, albeit not pathogenic *sensu stricto*, can cause seed decay of *S. hermonthica* by extracellular xylanases, pectinases, and amylases [16]. Interestingly, the implications of such findings can also be translated into the development of new control strategies that target the seed bank in highly infested and abandoned field sites. Despite some studies investigating the potential use of viruses to control weeds [17], their efficacy in controlling RPWs remains to be explored.

### Antagonism via secondary metabolites and volatile organic compounds (VOCs)

Recent high-throughput screenings of chemical libraries have led to the discovery of several compounds that interfere with strigolactone signalling. These include compounds inducing the germination of *S. hermonthica*, such as sphynolactone-7 [18^••^], inhibiting a strigolactone receptor in *S. hermonthica*, such as soporidine [19] and simple β-lactones [20], or inhibiting receptors of strigolactones from a range of other plant species, such as derivatives of *N*-phenylanthranilic acid [21]. Soil and plant-associated microbes can make structurally similar compounds. For example, bacterial strains of the genera *Streptomyces* and *Arthrobacter* produce anthranilic acid derivatives. Also, β-lactone derivatives are produced by bacteria and fungi, such as hymeglusin by *Fusarium*, obafluorin by *Pseudomonas fluorescens*, lipstatin and belactosins by *Streptomyces* spp. [22]. Other fungal metabolite classes that hold potential to suppress RPWs include sesquiterpenoids, tricothecenes (e.g. HT-2 toxin, neosolaniol, nivalenol, roridin A and verrucarins A, B, M), in addition to amino acid overproduction as highlighted above [12]. Tricothecenes are broadly distributed across the fungal genera *Fusarium* and *Myrothecium*, which are well-known RPW antagonists. As strigolactones are sesquiterpene lactones, it would be interesting to investigate if the observed suppressive effect of tricothecene-producing fungal RPW antagonists can be explained, in part, by competition for binding sites of the strigolactone receptors. Plant-associated strains from a range of bacterial genera, such as *Streptomyces*, *Azospirillum*, *Pseudomonas* and *Rhizobium,* have been tested for activity against RPWs. In most of these studies, however, the underlying mechanisms and metabolites were not characterized in detail. Nevertheless, a small lipophilic compound [23] and a small peptide [24] of *Azospirillum brasilense* were implicated in germination arrest of *S. hermonthica* and *P. aegyptiaca*, respectively.

A separate class of microbial metabolites for RPW control are the volatile organic compounds (VOCs). VOCs are chemically diverse small molecules with low vapour pressure that can, from a distance, regulate plant growth and root development [25]. The best example of a microbial VOC that can trigger suicidal germination of RPW seeds is ethylene [26]. Ethylene was successfully used as a soil fumigant to eradicate *Striga asiatica* in North and South Carolina [27], but this technology is not easily applicable in developing countries due to high costs and non-target effects on soil (micro-)biology. Alternatively, there is a high number of microbes able to produce ethylene. For example, ethylene produced *in vitro* by *Pseudomonas syringae* pv. *glycinea* [26] and *Klebsiella* sp. [28] induced seed germination of several *Striga* species, including *S. aspera*, *S. hermonthica* and *S. gesnerioides*. In addition, the sulphurous microbial VOC dimethyldisulfide produced by various bacterial genera such as *Burkholderia* [29] was implicated in *P. aegyptiaca* control [10]. Collectively these studies exemplify that soil and root-associated microbiomes hold a yet-untapped metabolic repertoire to *(1)* induce RPW seed germination in the absence of its host [30], referred to as suicidal germination, *(2)* suppress RPW seed germination, or *(3)* hinder the development of radicles and/or haustoria [12].

### Disruption of host-parasite signalling

Since seed germination and haustoria formation are crucial steps in the infection process of RPWs, it is interesting to explore the capability of soil and root-associated microbes to interfere with or disrupt this chemical signalling cascade. For example, after growing bacterial epiphytes from sorghum seeds in sorghum root exudates, the induction of *S. hermonthica* germination by the root exudate decreased almost completely and a reduced number of *Striga* attachments to the host root was observed. These findings were, to some extent, related to changes in the composition of phenolic compounds in the exudates [31]. In another example, when fungal strains (i.e. *F. oxysporum, Fusarium solani, Botrytis cinerea, Trichoderma harzianum*) were grown in liquid culture, the germination stimulants strigol, 5-deoxystrigol, 4-deoxyorobanchol, and the synthetic analogue GR24 were significantly degraded [32]. A myriad of signalling molecules (e.g. sterols, isothyacyanates, organic acids) that can induce RPW seed germination and haustorium formation are released in the root-soil interface. Because of antimicrobial properties [33], several of these signalling molecules may also indirectly affect RPWs via changes in the composition and activity of plant-associated microbial communities or via affecting the association with AMF. Although microbe-mediated chemical modifications or degradation of signals seem to work effectively in *in vitro* assays, the efficacy *in planta* as well as the impact on the mutualistic interactions between the plant and symbionts, such as AMF, are still underexplored areas of research in microbe-mediated RPW control.

## Indirect modes of action

### Enhancement of host nutrient acquisition

Exudation of strigolactones is induced by phosphorous (P) and, to some extent, by nitrogen (N) starvation [34], resulting in a ‘nutrient-dependent strigolactone negative feedback’. In other words, when a host plant is nutrient starved, it will start recruiting AMF via increased strigolactone exudation, which are then hijacked by RPWs as a signal of host presence. In line with this, exudates of P-starved tomatoes induced higher *Phelipanche ramosa* germination [35], however, when plants were colonised by AMF the biosynthesis of strigolactones was halted [36]. Moreover, some AMF were shown to increase root nodulation [37], which can improve both P and N uptake. This finding is particularly relevant for leguminous host plants of *Striga gesneroides* and *O. crenata*. Chemical fertilization (P and N) can negatively affect *S. hermonthica* germination, attachment and emergence [38]. Therefore, microbe-mediated provision of the host with labile sources of P and N is a potential mechanism that, indirectly, hampers the signalling between host and RPWs. Since AMF also depend on strigolactones to initiate symbiosis, working towards P provision via AMF association may not be a viable option as they might be outcompeted by RPWs. However, different strigolactone exudation profiles were observed for maize cultivars resistant and susceptible to *S. hermonthica*, dominated by sorgomol and 5-deoxystrigol, respectively. These exudates differentially affected seed germination of *S. hermonthica* and only minimally influenced AMF colonization [39^•^]. These findings point towards a need to better understand the specificity of distinct strigolactones on AMF symbiosis and RPW infections [40]. Apart from the well-known benefits of AMF, various other fungal and bacterial genera are effective P-solubilizers, through the production of organic acids such as citric, lactic and oxalic acids [41]. These include the fungi *Fusarium*, *Trichoderma*, and *Myrothecium,* and a wide range of bacteria such as *Pseudomonas*, *Streptomyces*, *Burkholderia¸* and *Rhizobium* — all of which have been linked to suppression of various RPWs. For these other fungi, however, the link between P-solubilisation and reduced RPW infection has not yet been established.

### Modulation of root physiology

Root-associated microbes can modulate root physiology and exudation both quantitatively and qualitatively [42,43]. For instance, upon AMF (*Glomus intraradices*) colonization of tomato, the level of strigolactones in exudates (i.e. solanacol, didehydro-orobanchol) was significantly reduced, resulting in lower seed germination of *P. ramosa* [36]. Whether this effect is indirectly caused by phosphate nutrition or directly via AMF colonization was not resolved in this study. Microbes may also modulate other root exudates with allelopathic properties that influence RPWs. An example is the sesquiterpene inuloxin C from the medicinal composite plant *Inulaviscosa* (syn. *Dittrichia viscosa* Greuter), which was shown to hinder seed germination of *P. ramosa* and several *Orobanche* species, even in the presence of strigolactones [44]. Other examples are the rye-cyanatines from cereals, which had an adverse effect on broomrape germination and development [45], and 6-chloroacetyl-2-benzoxazolinone, a derivative of 2-benzoxazalinone described as inhibitor of germination and radicle development of *O. crenata* [46]. Moreover, root exudation can also be influenced by aboveground pathogens and herbivores leading to changes in the composition and activity of root-associated microbes [47, 48, 49]. In addition to changes in root exudation, microorganisms can also induce changes in root architecture [50,51], and possibly root tissue distribution and chemical depositions (e.g. callose, suberin and phenolic compounds) that can act as physical barriers to RPW infections [52]. For example, the AMF *Gigaspora margarita* was shown to induce lateral root formation in *Lotus japonicus* via exudates and volatiles emitted from germinating spores [51]. Such shifts in root architecture can potentially lead to variation in RPW infection sites. For instance, it was shown that *O. cumana* had a preference for infecting younger thinner roots of sunflower, likely due to increased lignification of older root tissues [53]. It is noteworthy, however, that in these experiments it is challenging to disentangle microbe-induced effects on root chemistry from plant responses to RPWs and/or to the local environment.

### Induced systemic resistance

Several root-associated microorganisms can induce systemic resistance in plants against root and leaf pathogens [54]. Induced resistance responses are accompanied by substantial transcriptional changes in plant defence pathways, in particular salicylic acid and jasmonic acid, as well as changes in physiology and cell wall chemistry [54]. Recent studies have shown that salicylic acid and to some extent jasmonic acid signalling pathways can also be important for defence against parasitic plants [reviewed in Ref. 55]. When inoculated onto pea roots challenged with *O. crenata*, *Rhizobium leguminosarum* led to the induction of several defence-related enzymes and metabolites such as polyphenoloxidase, H_2_O_2_, lipoxygenase and the phytoalexin pisatin [56]. Similarly, *Streptomyces enissocaesilis* triggered polyphenoloxidase in sunflower, the host of *O. cumana* [57]. Although microbes can induce defence responses in multiple plant species that are hosts for RPWs, the underlying signal-transduction pathways and their conclusive role in suppression of RPWs have, to our knowledge, not yet been resolved.

## Outstanding questions and concluding remarks

Despite the mounting examples of soil and root-associated microbes influencing the life cycle of RPWs, there is still a scarcity of information on the underlying mechanisms by which these microbes operate. Moreover, many other outstanding questions remain to be answered. For example, what is the frequency of RPW-pathogenic and antagonistic microorganisms in the plant root microbiome? And, what is the impact of RPW infection on the host microbiome composition and antagonistic activity? In this context, there are a few intriguing recent studies. For example, it was shown that *Orobanche* and *Phelipanche* infections led to a significant decrease of microbial cell densities in the rhizosphere of parasitized plants [58]. Furthermore, two studies found that upon infection of tomato plants by *Phelipanche aegyptiaca* [59^••^] and of *Nitraria tangutorum* by *Cynomorium songaricum* [60], the endophytic microbiome (bacteria [59^••^] and fungi [60]) became more similar between the parasite and host plants. Hence, one may speculate that RPWs can, to some extent, modulate the host microbiome systemically and likely at the infection sites for their own benefit. Because of the physical connection of RPWs with their host through their vascular systems, this also enables the exchange of antagonistic microbes (e.g. endophytes) and compounds from the host to the RPW.

To date, most studies on microbe-RPW interactions focus on single microbes. However, the use of single members of the plant microbiome has proven to be an inconsistent strategy, particularly in field settings. Hence, designing functional synthetic microbial communities (SynComs) [61^•^,^62^] may be the way forward to more consistently suppress RPWs. To this end, the design should involve microbes with complementary modes of action (Figure 1) that act together or synergistically, and preferably at different stages of the parasite’s life cycle. In line with that, Oyserman *et al.* [63] recently introduced the concept of microbiome-associated phenotypes (MAPs), where modular microbiomes are engineered in concert with the host genotype to increase the efficacy of the desired trait. This reinforces the need to understand how each ‘module’ (or trait) behaves across different conditions, that is, the ecological context of trait function. Moreover, a microbial-mediated strategy for RPWs control should also take into account other commonly used agricultural practices (such as the use of organic amendments [64]), for instance by promoting the selective enrichment of microbes/SynComs with RPW suppressive functions. Current agricultural management practices used to control RPWs (e.g. crop rotation, trap/catch cropping) do not take into account the untapped importance of the microbiome. Considering the largely unexplored potential of microbiomes indigenous to the geographic regions where RPWs cause major crop losses, these microbiome-based strategies hold promise for developing and integrating novel and sustainable strategies for RPW control.

## References and recommended reading

Papers of particular interest, published within the period of review, have been highlighted as:

• of special interest
•• of outstanding interest

## Declaration of Competing Interest

The authors declare that they have no known competing financial interests or personal relationships that could have appeared to influence the work reported in this paper.
